# Strings and topological defects govern ordering kinetics in endothelial cell layers

**DOI:** 10.1038/s41567-025-03014-4

**Published:** 2025-10-09

**Authors:** Iris Ruider, Kristian Thijssen, Daphné Raphaëlle Vannier, Valentina Paloschi, Alfredo Sciortino, Amin Doostmohammadi, Andreas R. Bausch

**Affiliations:** 1 Heinz Nixdorf Chair in Biophysical Engineering of Living Matter, Garching, Germany; 2 Center for Functional Protein Assemblies (CPA), Technical University of Munich, Garching, Germany; 3 Center for Organoid Systems (COS), Technical University of Munich, Garching, Germany; 4 Matter to Life Program, Max Planck School, München, Germany; 5 Niels Bohr International Academy, The Niels Bohr Institute, University of Copenhagen, Copenhagen Ø, Denmark; 6 Department of Vascular and Endovascular Surgery, Klinikum rechts der Isar, Technical University of Munich, Munich, Germany; 7 German Center for Cardiovascular Research DZHK, Partner Site Munich Heart Alliance, Berlin, Germany

**Keywords:** Topological defects, Biological physics

## Abstract

Many physiological processes, such as the shear flow alignment of endothelial cells in the vasculature, depend on the transition of cell layers between disordered and ordered phases. Here we demonstrate that such a transition is driven by the non-monotonic evolution of nematic topological defects in a layer of endothelial cells and the emergence of string excitations that bind the defects together. This suggests the existence of an intermediate phase of ordering kinetics in biological matter. We use time-resolved large-scale imaging and physical modelling to analyse the non-monotonic decrease in the number of defect pairs. The interaction of the intrinsic cell layer activity and the alignment field determines the occurrence of defect domains, which defines the nature of the transition. Defect pair annihilation is mediated by string excitations spanning multicellular scales within the cell layer. Our results, therefore, suggest a mechanism by which intermediate ordering and string excitation might contribute to regulating morphogenetic movements and tissue remodelling in vivo.

## Abstract (web summary)

Imposing shear flow on a cell layer induces an ordering transition. Now it is shown that an intermediate phase of ordering occurs driven by an interplay between cellular activity and the aligning field.

## Main

Since 1858, the relationship between a homogeneous endothelium and blood flow patterns has been recognized, with endothelial cells displaying high levels of alignment in the aorta, in contrast to their misalignment at bifurcation points^1,2^. Subsequent in vitro studies revealed that endothelial cell layers have an intrinsic ability to realign and elongate in response to shear stress^3–5^. Since then, the extent of this tissue-wide global order has been considered a defining characteristic of various cell layers, influencing processes such as vascularization^6^, tissue regeneration^7^, development^8^ and morphogenesis^9–11^. However, it is increasingly evident that disrupting the order in localized areas, known as topological defects, serves as a hotspot for biological activity such as the regulation of cell apoptosis^12^, cell layer homeostasis and stem cell accumulation^13–15^. In non-living systems, it is well established that local defects are crucial for transitions between disordered and globally ordered phases^16^. Understanding those transitions has provided the basis for applications in materials science, ranging from superfluidity and melting^17–19^ to superconductors^20,21^. Yet, transitions between local and global order in living, cellular systems remain largely unexplored, partly due to the large length scales and timescales involved^22^. Instead, our understanding remains limited to characterizing the steady-state properties of disordered or ordered cellular assemblies. This lack of knowledge about intermediary states between disordered and ordered states of cell organization leads to heterogeneous and sometimes contradictory observations, as seen in endothelial cell alignment and de-alignment^23–27^. To unravel the kinetics of shear-flow-driven ordering in primary endothelial cells, we used time-resolved large-scale imaging paired with physical modelling. We show that the flow-induced order transition in endothelial cell layers is governed by a three-stage process in which the ordering kinetics is disrupted by an intermediate stage at which cells temporarily misalign, and multicellular defect strings emerge. We further demonstrate that an intricate interplay between the external shear flow and the activity of the living endothelial cell layer governs the emergence of defect strings and the concomitant intermediate ordering phase.

## Ordering kinetics characterized by three distinct phases

**Fig. 1:**
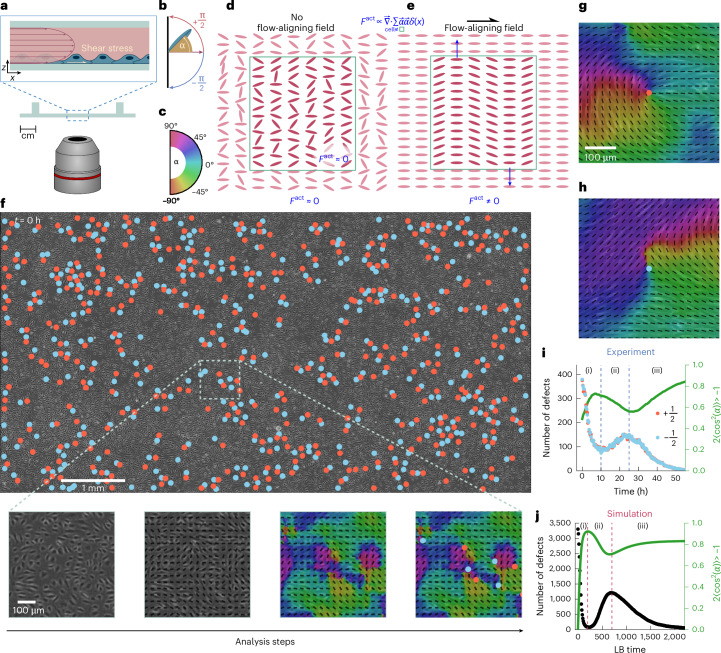
Shear-flow-induced alignment and defect dynamics in endothelial cell monolayers. **a**, Cells experience a shear flow along the *x*–*z* plane. **b**, Cell alignment is captured by the nematic director field, where the orientation *α* = 0 is defined with respect to the direction of shear flow. **c**, Nematic director is colour coded to visually represent cell alignment. **d**, Without a flow-aligning field, intercellular forces are randomly oriented and cancel each other out. **e**, Shear in the *x*–*z* plane acts as an affine alignment field, inducing elongation and ordering of the cells along that direction. As local ordering is established, intercellular forces stop cancelling out, resulting in mesoscopic active forces *F*^act^ (collective active stresses), approximated as extensile forces (*ζ* > 0)^68,69^. **f**, Field of view of the endothelial cell monolayer during a flow experiment. The zoomed-in views show a phase contrast image of a cellular monolayer and simultaneously illustrate the analysis steps with increasing detail, containing the nematic director field, orientation field and nematic topological defects. **g**, Representative +1/2 defect indicated by a red dot. **h**, A representative 1/2 defect indicated by a blue dot. **i**, Number of defects and the average global nematic ordering of the endothelial cell monolayer during the alignment process in the experiments. The light green area indicates the 95% confidence interval. The average global nematic order was computed by measuring the local order parameter at 53,607 evenly distributed points within the endothelial cell monolayer. The 95% confidence interval is barely visible because it is comparable in width to the plotted line. **j**, Number of defects (black dots) and the global nematic ordering of the endothelial cell monolayer during the alignment process in the lattice Boltzmann (LB) simulations.

To gather sufficient statistical data to fully describe the phase ordering kinetics between disordered and ordered cellular organization, we examined the effect of a constant superjacent shear flow (~20 dyn cm^−2^) on a confluent layer of human aortic endothelial cells (HAOECs)^5,28,29^ with an automated microscope setup, which allowed the stitching of high-resolution images on a scale of several millimetres during periods of up to 64 h (Fig. 1a). The shear rate was chosen to mimic physiological conditions. Experiments were performed independently six times at high densities, all of which showed non-monotonic coarsening.

**Extended Data Fig. 1:**
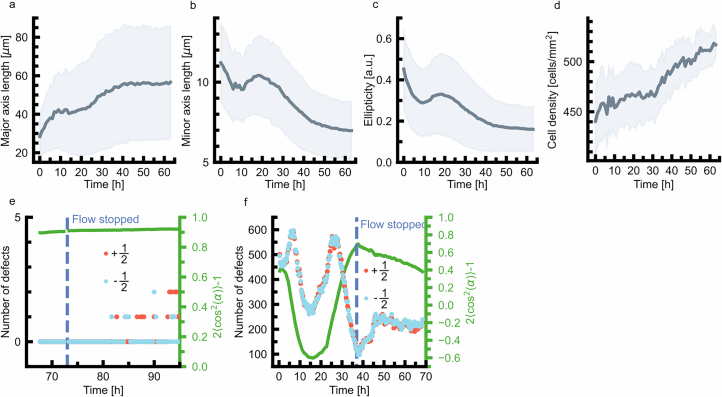
Direct effect of shear flow. (**a**) Average major axis length of HAOECs during exposure to shear flow. (**b**) Average minor axis length of HAOECs during exposure to shear flow. (**c**) Average ellipticity of HAOECs during exposure to shear flow. An ellipticity of 1 corresponds to a perfect circle. (**a**–**c**) Bright grey areas indicate the standard deviation. A minimum of 644 cells was evaluated per time point for all panels. (**d**) Cell density as a function of time. Increase in cell density over two days was approximately only 10%. The bright grey area represents the standard deviation. The average was computed by evaluating cell density in five distinct regions of 1.3 mm × 1.3 mm each. (**e**) Shear flow is stopped at different points of the experiments. As indicated by the blue vertical line in the panel, we stopped the flow when the system was almost perfectly aligned. (**f**) The flow was stopped during the steady-state ordering phase before the system was fully aligned. The light green shaded area represents the 95% confidence interval. The average global nematic order was computed by measuring the local order parameter at 53,928 evenly distributed points in (**e**) and 53,766 points in (**f**) within the endothelial cell monolayer. The 95% confidence interval is barely visible because it is comparable in width to the plotted line.

During HAOEC alignment and elongation (Extended Data Fig. 1), the emergence of nematic order was observed in the phase contrast images (Fig. 1b,c and Supplementary Video 1). To quantify the order, we extracted the local nematic director field of the endothelial cell monolayer in the full field of view (Fig. 1b,f). From this, we obtained the orientation angle *α*, colour mapped throughout the field of view to spatially resolve and visualize the alignment^30,31^. We identified the presence of nematic +1/2 and –1/2 defects within the cellular monolayer (Fig. 1c,f–h). On application of shear flow, an initially disordered layer of cells evolved into a well-aligned ordered layer, and the density of topological defects dropped substantially from over 400 defect pairs over the entire field of view of 40 mm^2^ in the initial state to around 0 at the final state (Fig. 1i). Although in passive materials, the ordering kinetics would be characterized by a monotonous decrease in the topological defect density^32^, here we observed three distinct phases driven by the non-equilibrium nature of the cell layer: in the initial ordering phase (i), exposure to shear stress induced the cells’ elongation and alignment along the flow direction. This was accompanied by a decrease in defect density in the first 9 h after flow was applied. In the subsequent intermediate disordering phase (ii), from *t* = 10 ± 2 and *t* = 29 ± 5 h, a transient reduction in cellular alignment and an increase in defect density was observed. In the final ordering phase, which we call the steady-state ordering phase (iii), the system realigned again, almost all nematic defects disappeared and a long-range orientational order was established (Fig. 1i and Supplementary Video 1). The observed defect densities emphasize the need of large-area observations to acquire sufficient defect statistics, by selecting smaller fields of view (<500 μm × 500 μm), large variability in the observed behaviour was observed. It required fields of view of larger than 500 μm × 500 μm to display consistent non-monotonic alignment dynamics.

## Interplay between local activity and global shear

The appearance of the intermediate disordering phase suggests that although shear flow drives the ordering of cells, the active motion and cell–cell interactions within the endothelial layer oppose the induced ordering. To test this, we simulated the transient behaviour using active nematic theory (see the ‘Simulations’ section). Here we used a coarse-grained model to solve the local velocity $\bf{v}$ and the nematic orientation tensor *Q*. The superjacent shear flow was modelled as an external aligning field that energetically penalizes orientations perpendicular to the flow direction, similar to an anisotropic quenching field^33^. Although we disregard the more complex effects of mechanosensitive shear flow sensing that cells may experience, this simplified approach was sufficient to induce a global nematic alignment within the cell layer. We modelled the activity of the endothelial cells by out-of-equilibrium force contributions, often called active force, with the lowest-order non-equilibrium dissipative terms, dipole forces, which are assumed to be homogeneous in space and aligned along the local elongation axis, which evolve with the nematic evolution equation (Fig. 1d,e). We adopted this particular model because of its simplicity, the precise mapping between non-equilibrium stresses in active matter systems and the stresses generated by collectively migrating endothelial cells across varying densities remains experimentally unresolved. These mechanical stresses not only influence the defect dynamics but also lead to increased cell shape deformation, leading to an increase in cellular ellipticity as the number of topological defects grows in active turbulence^34–36^.

**Fig. 2:**
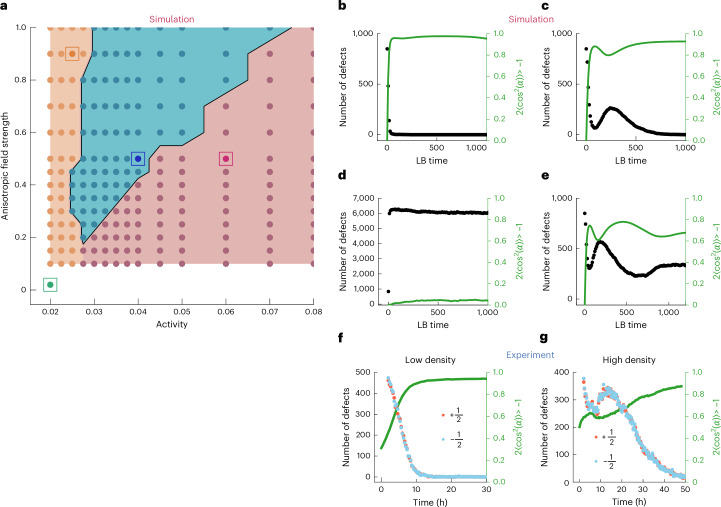
Interplay between cellular activity and external aligning field governs alignment dynamics. **a**, Phase diagram obtained from simulations shows that the non-trivial, non-monotonic coarsening behaviour, highlighted in blue, only exists within a very defined region. **b**, The system displays a monotonous alignment behaviour, highlighted in orange, for high anisotropic field strength and low activity within the nematic director field. **c**, In the blue region, the interplay between the anisotropic field strength and the intrinsic activity gives rise to a three-stage, non-monotonous alignment behaviour. **d**, When activity and flow anisotropic field are both low, the cells do not display localized order, resulting in an isotropic state, highlighted in green in the phase diagram. The number of defects saturates as the orientational order becomes ill-defined. **e**, When the activity is the dominating parameter, the system fails to align and evolves towards a state with only local nematic alignment, resulting in active turbulence, highlighted in red. **f**, At lower cell densities, the endothelial cells align monotonously along the flow direction, lacking the three-stage dynamics. The green line represents the average global nematic order, computed by measuring the local order parameter at 46,475 evenly distributed points within the endothelial cell monolayer. The light green area indicates the 95% confidence interval. The 95% confidence interval is barely visible because it is comparable in width to the plotted line. **g**, At higher cell densities, the endothelial monolayer displays non-trivial alignment dynamics characterized by a non-monotonous alignment. The green line represents the average global nematic order, computed by measuring the local order parameter at 54,249 evenly distributed points within the endothelial cell monolayer. The light green area indicates the 95% confidence interval. The 95% confidence interval is barely visible because it is comparable in width to the plotted line.

The activity and anisotropic field competed at different timescales^37,38^, which resulted in a variety of behaviours (Fig. 2), including a non-monotonic coarsening behaviour (Fig. 1j). As the cells elongated under the shear flow and local orientational order was established, the active stress from fluctuations in the orientation field was sufficient to nucleate topological defects and transiently destroyed the local orientational order in the system. Eventually, the orientation of the cells became almost perfectly aligned along the preferred axis set by the external field. When the shear flow is removed, the system starts to return to a disordered state (see the ‘Removal of shear flow’ section).

Although the experimental setup offered a limited capacity to explore the impact of gradually varying activity on this transition, simulations provided a more comprehensive understanding of how the activity of the monolayer^39^ and the strength of the external flow field influence the nature of the cell-ordering process. The phase diagram illustrates how the interplay between activity and the external field’s strength affects the kinetics of transition between disordered and ordered states (Fig. 2a). As expected for passive materials, in the absence of or at low values of active stresses, when the aligning field dominates on all timescales, the ordering kinetic follows a monotonic rarefaction of topological defects (Fig. 2b). By contrast, larger active stresses result in a defect-loaded steady state, called active turbulence (Fig. 2e). Only when the active stress and the external field compete on similar timescales (see the ‘Timescales’ section) can we recover the experimental behaviour, where the global nematic order parameter exhibits three distinct transient phases (Fig. 2c). We point out that for a weak anisotropic aligning field, the system remains in an isotropic state with no emergence of global order (Fig. 2b). The existence of a threshold shear stress, below which endothelial cells no longer respond to flow, has already been reported in the literature^26,40^ and the transition from this isotropic, disordered state to an ordered state through control of activity and an external field has been investigated, revealing the emergence of velocity lanes within the ordered state^41,42^. Therefore, only in an intermediate regime does the intricate interplay between activity-driven defect nucleation and flow alignment forces result in an intermediate increase in defect numbers. We note that it is possible for additional oscillations in defect density to occur in this intermediate ordering as defect pair nucleation and annihilating can be correlated in space and time^43^.

**Extended Data Fig. 2:**
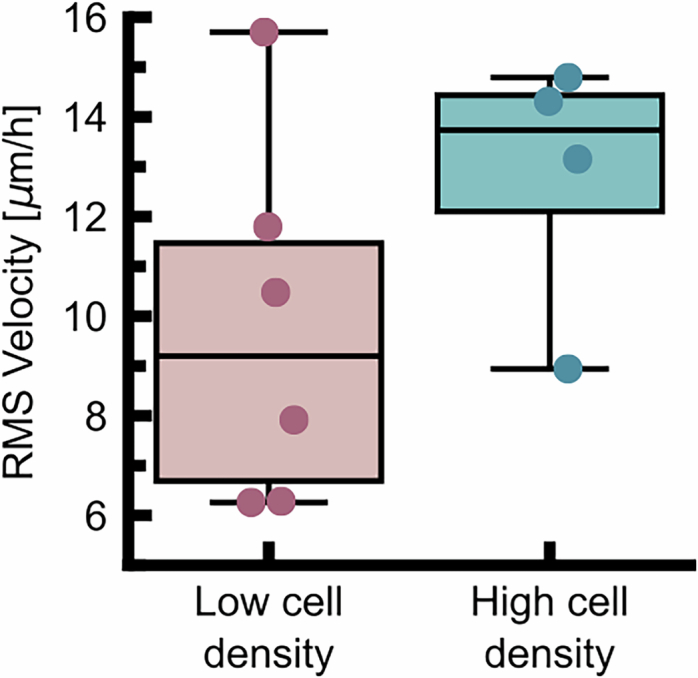
The average cell speed for different densities. The average cell speed for low (300-600 cells/*mm*^2^) (N=6) and high cell (600-1200 cells/*mm*^2^) (N=4) density. The center line of each box plot indicates the median, the box edges represent the interquartile range (IQR), and the whiskers extend to the furthest data points within 1.5 × IQR. Individual data points are overlaid as dots.

Although it was not possible to directly isolate the effect of activity in our experiments, we found that within the density ranges explored, increasing density had a similar impact on coarsening kinetics as increasing activity in the model (Fig. 2f,g). In particular, at low densities, experiments showed monotonic coarsening and the non-monotonic kinetics was only observed at high cell densities, where increased motility arose from cell–cell interactions (Extended Data Fig. 2). These densities remained relatively constant throughout the experiments due to limited proliferation (Extended Data Fig. 1d). Importantly, the reported densities were not high enough to cause cell arrest and immobility^44^. Further increasing cell density through seeding was not feasible, as the monolayer began to exhibit notable cell extrusion. Although resolving the direct relationship between activity and density remains an open question and is beyond the scope of this study as a variety of different coupling terms have been proposed^45–48^, the observed behaviour, along with negligible proliferation and increased monolayer velocity with rising density, suggests that within the explored density ranges for endothelial cells, increasing density could have a similar effect on coarsening kinetics to heightened activity of the monolayer.

## Anisotropic correlation lengths

**Fig. 3:**
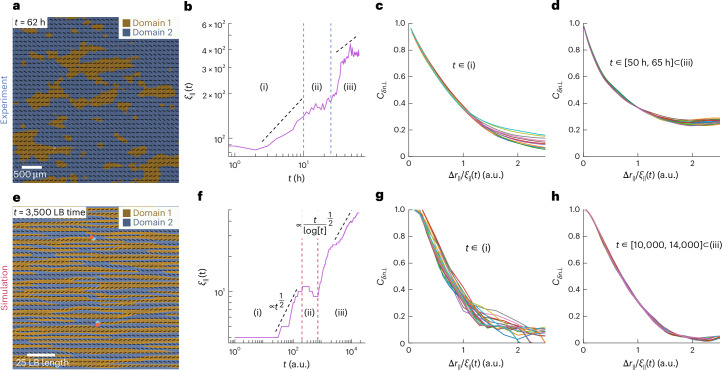
Three distinct scaling behaviours of domain growth. **a**, Illustration of the domains with positive or negative projection $\delta {n}_{\perp }$ values corresponding to the experimental data extending anisotropically along the flow axis. **b**, Correlation length *ξ*_∥_ exhibits distinct growth rates. Straight dashed lines indicate different regimes, identified based on extrema in defect densities. The correlation functions shown in **c** and **d** were computed by evaluating pairwise correlations at 524,508 points within the endothelial cell monolayer. The correlation length shown is derived from the average correlation function and represents a single realization in time. This behaviour is representative of other independent realizations. **c**, Correlation functions for the experimental data during the initial ordering phase (i). The length scale Δ*r*_∥_ is normalized by *ξ*_∥_. **d**, Correlation functions for the experimental data during the late stages (iii). The length scale Δ*r*_∥_ is normalized by *ξ*_∥_. **e**–**h**, Simulation figures corresponding to **a**–**d**, respectively. We note, however, that due to the limited amount of data in time, it is not feasible to perform a proper scaling measurement; the dotted and dashed lines in **b** and **f** are a visual guide to the eye.

**Extended Data Fig. 3:**
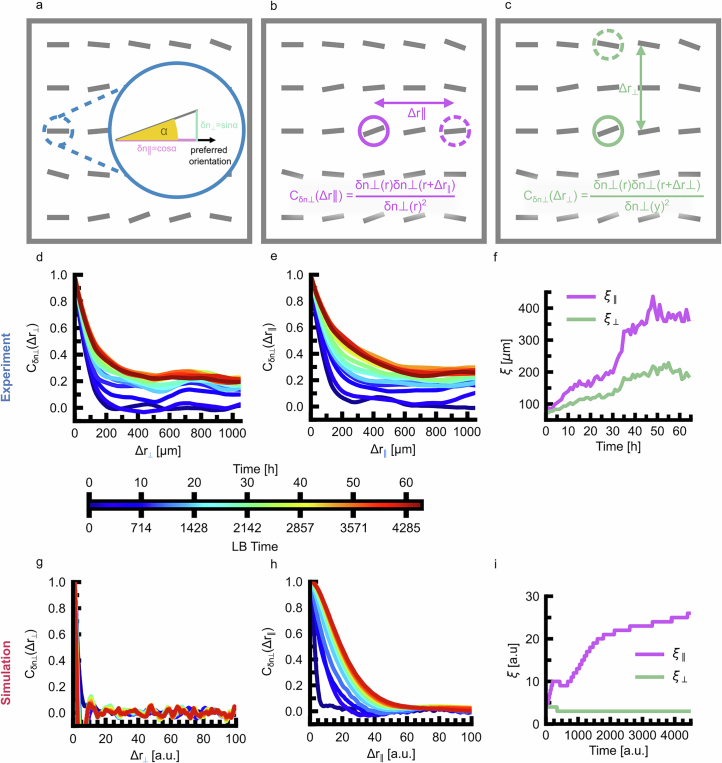
Correlation dynamics reveal anisotropic growth of similarly aligned regions. (**a**) Projections of the nematic director from the preferred axis of orientation $\delta \overrightarrow{n}$. (**b**) Anisotropic growth of correlation length parallel *Δ*r_∥_ and (**c**) perpendicular *Δ*r_⊥_ to the preferred axis. (**d**) Time evolution of the perpendicular *C*_*δn*⊥_(r_⊥_) and (**e**) parallel *C*_*δn*⊥_(r_∥_) correlation function for the experimental data, (**f**) with corresponding correlation lengths. The correlation functions were computed by evaluating pairwise correlations at 524,508 points within the endothelial cell monolayer. The correlation lengths in (**f**) are derived from the averaged correlation function and represent a single realization in time. The behavior shown is representative of other independent realizations. (**g**–**i**) Corresponding simulation figures to figures d-g.

Because of the preferred orientation axis set by the direction of the superjacent flow, the coarsening of the local nematic order was not isotropic. Rather, the system can be split into domains of negative and positive rejection to the preferred axis *δn*, which divides these domains (Fig. 3a,e). These domains are selected because the additional energy cost of the external field on the nematic orientation is identical for *δn*_⊥_ and –*δn*_⊥_. The multicellular orientationally ordered domains slowly coarsened in an anisotropic manner as global order was established (Supplementary Videos 2 and 3). This is best quantified by measuring the correlation functions ${C}_{\delta {n}_{\perp }}(\Delta {r}_{\parallel })$ and ${C}_{\delta {n}_{\perp }}(\Delta {r}_{\perp })$ that resolve the orientation correlation along the perpendicular (Δ*r*_⊥_) and parallel (Δ*r*_∥_) directions to the preferred axis (see the ‘Correlation functions’ section). The correlation lengths found from the correlation function along the parallel (*ξ*_∥_) and perpendicular (*ξ*_⊥_) axes to the preferred orientation showed asymmetric growth of similarly aligned regions in both model and experiments, where coarsening of the correlation length occurred primarily along the preferred axis (Extended Data Fig. 3a,e).

**Extended Data Fig. 4:**
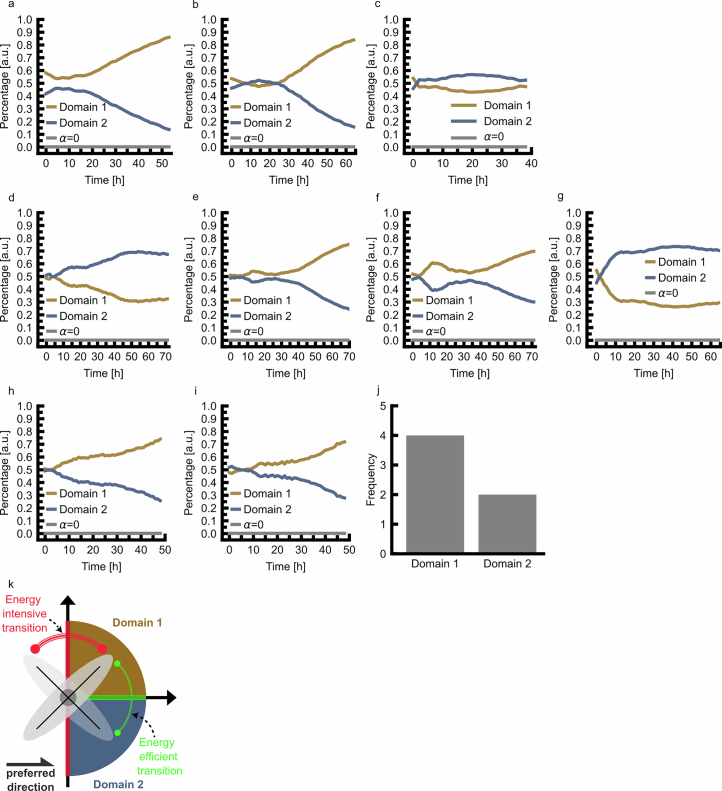
Growth dynamics of different domains for different experimental realizations. (**a**–**c**) Growth dynamics showing one domain dominating in time due to secondary active torque, quantified by looking at the average percentage of the system in domain 1 and domain 2, for the experimental realizations featuring monotonous alignment. (**d**–**i**) Growth dynamics for the experimental dynamics of the 6 cell densities which resulted in non-monotonic coarsening. (**j**) The frequency at which a preference for domain 1 or domain 2 was observed for the six experimental realizations that displayed non-monotonic coarsening. (**k**) Two domains (blue and brown) where the local director has the same energy cost due to the preferred orientation.

**Extended Data Fig. 5:**
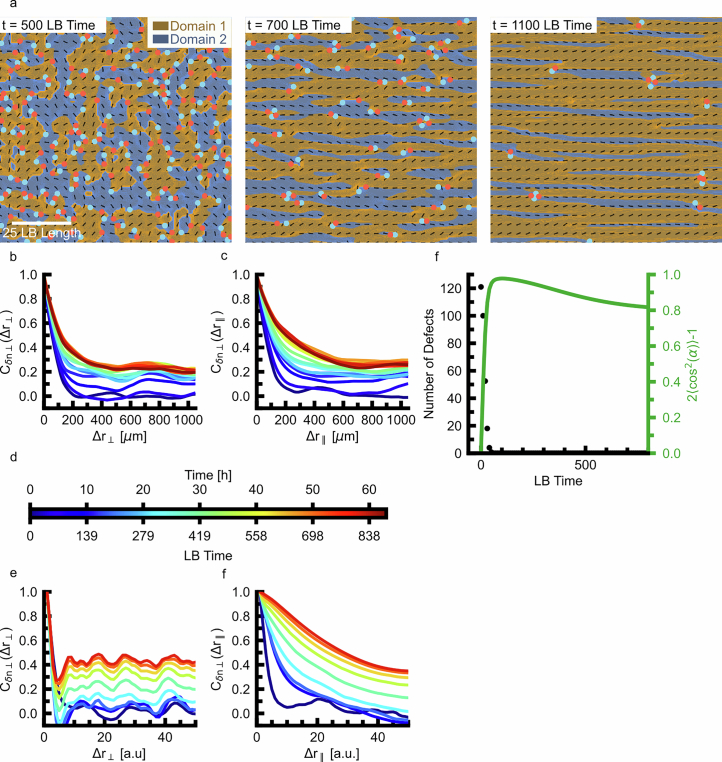
Simulations with additional self-generated spin. (**a**) Snapshots of the simulations with additional self-generated spin ∣*f*_*q*_∣ = 0.002. (**b**–**f**) The correlation functions of Extended Data Fig. 3 when we add a self-generated spin ∣*f*_*q*_∣ = 0.002 to our model. Figure b,c and d are the experimental data of Extended Data Fig. 3. (**g**) Monotonic coarsening kinetics is observed with self-induced spin alone ∣*f*_*q*_∣ = 0.002, but with active stress turned off *ζ* = 0.

Although simulations qualitatively capture the behaviour of correlation length parallel and normal to the preferred direction set by the flow, the coarsening dynamics along the perpendicular direction are less pronounced compared with the experimental measurements. This is due to a secondary effect of endothelial cells generating a spontaneous rotation^49^ (see the ‘Active torque’ section and Extended Data Figs. 4 and 5).

## Three-stage kinetic ordering

In the initial ordering phase (i), orientation correlation functions ${C}_{\delta {n}_{\perp }}$ along the preferred axis *r*_∥_ decayed to zero over time, and the characteristic correlation lengths *ξ*_∥_ increased exponentially (Fig. 3b). From the simple dimensional analysis of diffusive isotropic–nematic transitions^50^, the growth of *ξ*_∥_ is expected to follow *t*^1/2^, which is consistent with our simulation results and close to the experimental data (Fig. 3b,f). This suggests a negligible impact from defect annihilations on the ordering kinetic in this initial phase. As the correlation functions did not collapse with the distance normalized by the correlation length Δ*r*_∥_/*ξ*_∥_, the growth of ordering did not exhibit dynamic scaling behaviour, which would be expected for kinetic ordering in passive materials (Fig. 3c,g)^51^.

In the subsequent intermediate disordering phase (ii), the asymptotic value of the correlation function at long distances did not drop to zero. It reached a finite value that increased with time, indicating the appearance of a true long-range order (Extended Data Fig. 3e,h). The characteristic correlation length *ξ*_∥_ ceased to increase and remained stable whereas the number of defects rose, which is attributed to the increase in cellular forces as the cells begin to align. As the active forces reached a critical value, an active instability set that caused defects to nucleate^52^.

In the steady-state ordering phase (iii), the correlation functions converged to a finite value and entirely collapsed with Δ*r*_∥_/*ξ*_∥_, indicating a dynamical scaling of length scales as the system became more ordered (Fig. 3d,h). The characteristic correlation length *ξ*_∥_ increased again, and for the final times, this length scale is expected to follow the 1/2 power law with a logarithmic correction due to the interaction between topological defects^32,51^. This indicated that the coarsening in the final stage was dominated by long-range logarithmic interaction between defects^51^.

## Heterogeneous distribution of bound defect pairs

**Fig. 4:**
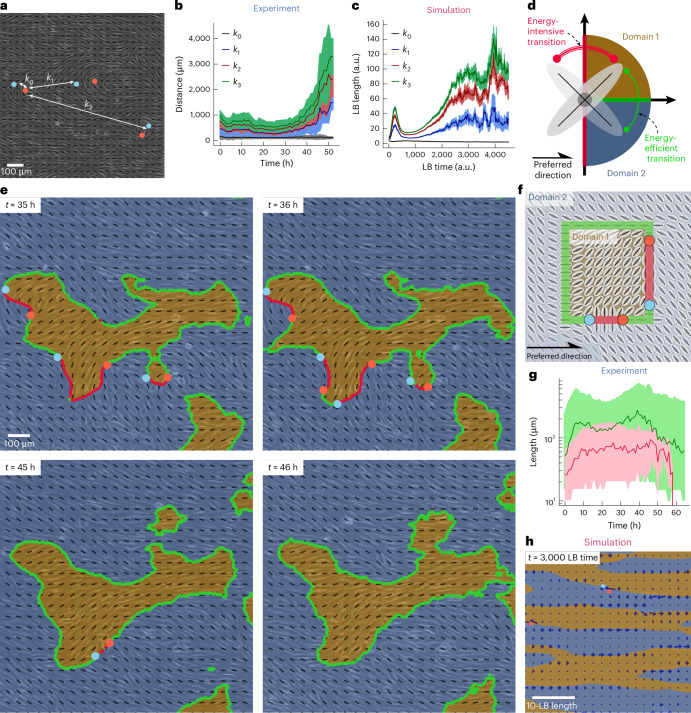
Nematic defects interact through strings. **a**, Distance to the *k*th nearest neighbour between two defects of opposite charge. **b**, Time evolution of the average distance between the *k*th nearest neighbours for the experimental data. The brighter areas indicate the standard deviation from the mean for the corresponding colour. A minimum of *N* = 4 defect pairs was used per frame. Fewer than ten defect pairs were available only for the last four time points, when long-range order emerged in the monolayer. **c**, Time evolution of the distance between the *k*th nearest neighbours for the simulation data. The brighter areas indicate the standard deviation from the mean for the corresponding colour. **d**, Two domains (blue and brown) in which the local director, uniquely defined within −π/2 ≤ *α* ≤ π/2, have the same energy cost due to the preferred orientation. Continuous transition from domain 1 to domain 2 can occur through either an energy-efficient (green) or energy-intensive (red) rotation. **e**, Nematic defect sitting on the domain walls interacts through strings with parallel orientation (green) and perpendicular orientation (red) to the preferred orientation axis. **f**, Defects remove the requirement for continuous director rotation, leading to alternating green and red segments of high- and low-energy-efficient regions. The orientation of the self-propulsion of the +1/2 defect depends on the position of the defect pair. Here two defect pairs are shown, one, aligned with the preferred axis, where self-motility eliminates perpendicular director alignment and another would increase perpendicular alignment. **g**, Median length of the strings with perpendicular orientation (red) decreases to zero as the system coarsens. The length of the green string initially increases and eventually plateaus during coarsening for experimental data. The brighter areas illustrate the interquartile range for data of the corresponding colour. The length of at least *N* = 370 green strings was measured per frame. The length of at least *N* = 5 red strings was measured per frame for all data points, except for the final one, where *N* = 2. Fewer than ten red strings were available only during the final four time points, when long-range order had emerged in the monolayer. The length of at least *N* = 5 red strings was measured per frame for all data points, except for the final one, where *N* = 2. Fewer than ten red strings were available only during the final four time points, when long-range order had emerged in the monolayer. **h**, Force field of the nematic director field. The forces (blue arrows) are the highest along the domain boundaries.

**Extended Data Fig. 6:**
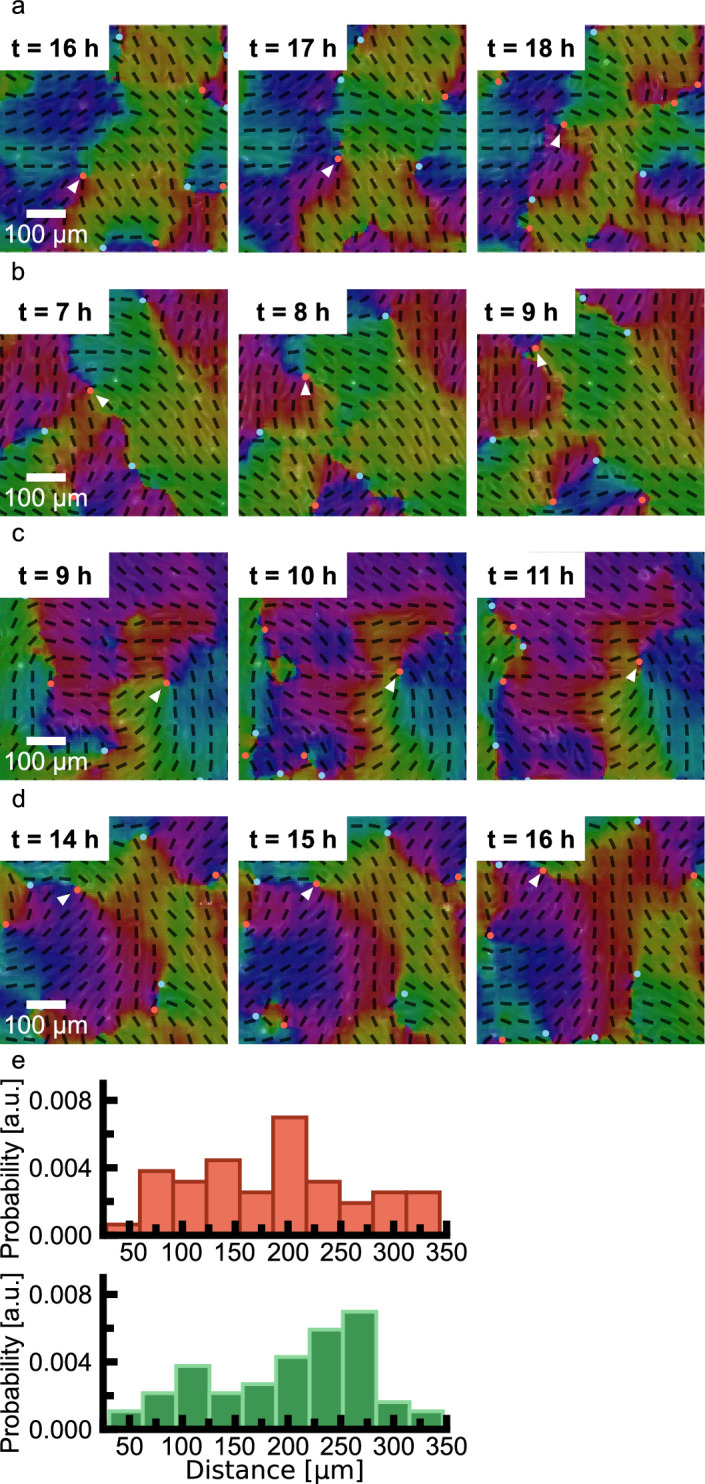
Additional defect statistics. (**a**–**d**) The motion of different + 1/2 defects (shown as red circles) in the absence of flow showing that they move in the direction consistent with an extensile active nematic. (**e**) Probability of finding the nearest defect at distance at a distance from a cell division event (top) and a random point in the cell layer (bottom). For each condition more than 50 division events were analyzed.

**Extended Data Fig. 7:**
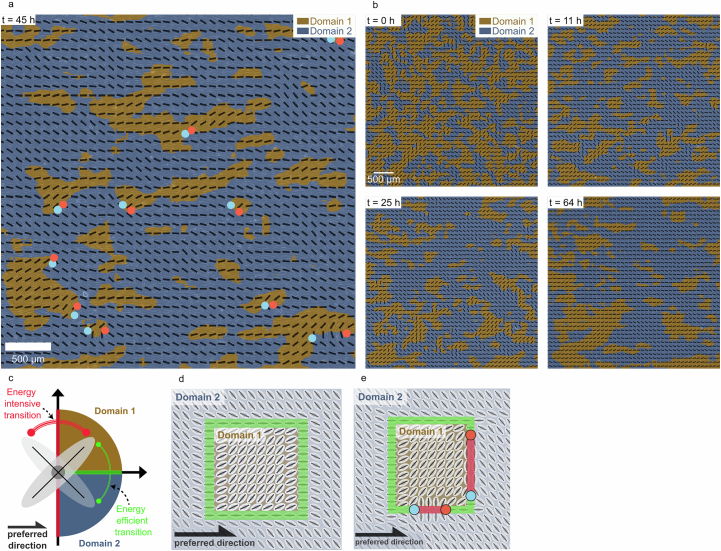
The behaviour of the domain boundaries. (**a**) In the experimental data, the nematic defects sit on the domain boundaries. The nearest-neighbor pairs remain at a close distance from each other, while the distance to the second nearest neighbor increases during coarsening. (**b**) Domains start symmetrically spaced, but over time, they coarsen anisotropically. The coarsening of the domains in the experimental data halts (t=11h to t=25) during the intermediate disordering phase (II) and progresses during the initial ordering phase (I) (t=0 to t=10h) and the steady-state ordering phase (III) (t=26 to t=64h), during which domains become more anisotropically shaped. (**c**) Two domains (blue and brown) where the local director has the same energy cost due to the preferred orientation. Transitioning from Domain 1 to Domain 2 can occur via an energy-efficient (green) or energy-intensive (red) rotation. As the nematic field continuously rotates, boundaries emerge where the director is either most (green, parallel) or least (red, perpendicular) energetically favored to align with the flow-aligning field. (**d**) This represents the lowest-energy configuration connecting the two domains. (**e**) Introducing defects removes the requirement for continuous director rotation, leading to alternating red and green segments. The orientation of the velocity of + 1/2 defect depends on the defect pair’s position. Along the x-axis, the motion eliminates perpendicular director alignment, annihilating the defect pair and restoring the configuration in (**b**). Along the y-axis, however, the motion reinforces the disfavored perpendicular alignment, creating a competition between self-propulsion and misalignment with the flow-aligning field, ultimately stabilizing the defect pair.

The emergence of three-stage ordering kinetics, linked to the non-monotonic evolution of topological defects during the establishment of long-range orientational order (Figs. 1i,j and 3b,f) prompted us to investigate the spatiotemporal defect dynamics leveraging the extensive field of view. A detailed examination of defect dynamics revealed that the emergence of new defect pairs was highly localized at the onset of the defect nucleation phase. During this phase, multiple defect pairs nucleated in close proximity to one another, whereas a substantial proportion of the field of view remained defect free. We characterized this heterogeneity by analysing the distance between the *k*th nearest neighbours of oppositely charged defects (Fig. 4a)^53^. We observed in both experiment and simulation that after the defect pairs nucleated, they did not move further apart as expected in traditional active turbulence (Extended Data Fig. 6a–d) nor did they immediately annihilate, as seen previously in flow-tumbling active systems with external fields^33^. Instead, the defects remained relatively bound to each other (Fig. 4b,c; *k*_0_). Hence, in contrast to a homogeneous increase in defect spacing, we identified regions densely populated with defects interspersed with void areas in which no defects were present (Extended Data Fig. 7a). This heterogeneity in the spatial defect distribution impacted the later stages of ordering kinetics. Consequently, during the final coarsening, the distance between the nearest-neighbour defect pairs *k*_0_ did not increase. Instead, the distance of the higher-order pairs *k*_*i*>0_ increased as the distance between defect-rich regions increased (Fig. 4b,c; *k*_*i*>0_). Eventually, at late times, the logarithmic interactions between the separate defect pair configurations became dominant, and all topological defect pairs annihilated. Even as the number of defects decreased and the domains grew larger (Extended Data Fig. 7b), we did not observe a notable change in the nearest-neighbour distance between oppositely charged defects. This is because defect pairs tend to cluster in groups, and only these groups become increasingly sparse. This unique behaviour did not occur in the absence of activity or the external flow field, indicating an intricate interplay between the cell activity, aligning field, domain interfaces and the topological defects residing on them.

## String excitations

In the absence of an external field, it is well established that activity unbinds pairs of oppositely charged nematic topological defects^52,54^. Interestingly, in both experiments and simulations, we measured a fixed, small distance (~123 ± 11 μm, equivalent to up to 11 cell sizes) between nearest-neighbour *k*_0_ oppositely charged ±1/2 defects throughout the ordering process. This indicates that despite the intrinsic activity of the cells, the oppositely charged defect pairs remained bound during coarsening, even after the nucleation phase. We hypothesized that the emergence of such stable defect configurations is governed by topological strings that bind defect pairs together^55^, which are driven by the interplay of active stresses within the cell layer and the external aligning field (Fig. 4d). This is because the additional field changes the energy level of the different nematic orientations. In a conventional nematic, the director is uniquely defined over a 180° range in vector space, maintaining rotational symmetry. Consequently, all orientations are equivalent, and defining this range from 0 rad to π rad, –π/2 rad to π/2 rad, or –π/4 rad to 3π/4 rad would typically be arbitrary. However, the external field breaks this rotational symmetry and preserves mirror symmetry: ±*δn*_⊥_ remain identical. This makes the choice of director orientation non-trivial. We define two domains with equal energy (vector quadrants one and four) and set the director range to –π/2 rad to π/2 rad (Fig. 4d). This choice of domains ensures identical energy costs. By contrast, non-trivial divisions along random angles relative to the preferred axis (*x* axis here) would result in domains with different energy levels. In defect-free systems, a smooth rotation must connect different orientations. Transitioning from domain 1 to domain 2 requires passing through either *δn*_⊥_ = 0 (lowest energy, green lines) or *δn*_⊥_ = ±π/2° (highest energy, red lines). These correspond to isophase lines, which, unlike in standard nematics, are energetically distinct due to mirror symmetry. This is similar to domain walls in magnetic systems, where green isophase lines represent the lowest energy and predominantly enclose domains in defect-free configurations (Extended Data Fig. 7c–e). The red walls are unfavoured because of the high energy cost, analogous to Zeeman splitting, and motivates our comparison with the pair superfluid behaviour in which the nematic symmetry of boson pairing interacts with the preferred direction imposed by the Zeeman field^56^ (see the ‘Effective Zeeman splitting of strings’ section).

**Extended Data Fig. 8:**
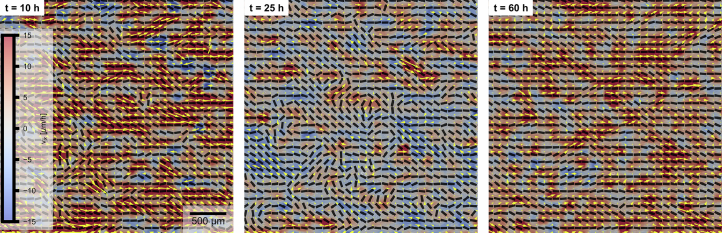
Snapshots of cell motion. Snapshots from Supplementary Video 7.

When a defect pair is introduced, the director reorientation is no longer smooth. The string structure fragments into regions of alternating red and green segments, with sharp orientation changes at defect positions. The high-energy red regions induce an additional attraction between defects due to the additional energy cost, potentially counteracting the unbinding energy^52^ and resulting in stationary defect pairs (Fig. 4f). As a result, string excitations stabilized the oppositely charged defect pair configurations until the final ordering phase. This stabilization causes the defect pairs to become long-lived. Experiments confirm that long-lived defect pairs exhibit this type of orientation and indeed live on the strings that we identify, further showcasing their physical importance. Consequently, defect motility was highly localized to movement along the strings, the strings provided a path for the annihilation of defects (Supplementary Videos 4 and 5), and the strings that lined up perpendicular to the preferred axis (Fig. 4g; red) eventually decayed as defect pairs reoriented, allowing the elastic interaction to overcome the self-motility and resulting in annihilation at later times. Meanwhile, the strings extending along the preferred axis (Fig. 4g; green) increased during the initial coarsening, but eventually, the average string length appeared to remain constant as the energy costs of the domains were identical. Examining snapshots of the ordering kinetics revealed that eventually, long strings extending along the preferred axis dominated the system at late times (Supplementary Videos 4 and 5). The large extent of these strings is remarkable as they potentially provide a path for the long-range transmission of mechanical forces. Although force measurements were not available for the experiments, we could probe this mechanism in the simulations. Indeed, simulations confirmed that the strings act as hotspots for focusing active forces (Fig. 4h) as these string-like structures act as guides for splay deformations, which, in turn, concentrate active stresses and lead to prominent cell displacements both with and against the background shear flow (Supplementary Video 7 and Extended Data Fig. 8). This results in a coupling between orientation and velocity similar to what was previously reported in bacterial systems within patterned environments^57^ or in microtubule–kinesin mixtures confined to patterned surfaces^58^.

In summary, the high temporal and spatial resolutions achieved in our experimental setup allowed us to describe the ordering kinetics of endothelial cell layers within the framework of active nematic liquid crystals, highlighting the governing role of topological excitation and explaining endothelial cell alignment as a dynamic transition with cellular misalignment as an intermediate stage. We note that although changes in cell shape could affect phenomenological parameters, our main experimental results were captured without incorporating any shape- or time-dependent modifications. This demonstrates the robustness of our framework, showing that transient dynamics can naturally emerge from the interplay of timescales. The introduced unifying framework reconciles previous contradictory observations reporting perpendicular alignment already at physiological shear stress levels^26,59^, in contrast to previous observations that linked perpendicular cell alignment to elevated pathological shear stress levels^25,27^. Our ability to capture the full statistics of millimetre-sized samples shows that cellular alignment is not strictly monotonic and can involve transient disalignment phases, even at physiological shear stress levels. The importance of the transient regime in endothelial cell alignment reveals the need for precise temporal and spatial resolutions in detecting changes in protein expression, exemplified by the previously observed transient upregulation of JNK2 in bovine aortic endothelial cells^60^. A limitation of current experiments is the inability to decouple cell activity from density without disrupting the endothelial mechanosensing of flow. Future studies could overcome this by using molecular strategies to modulate mechanotransduction pathways.

**Extended Data Fig. 9:**
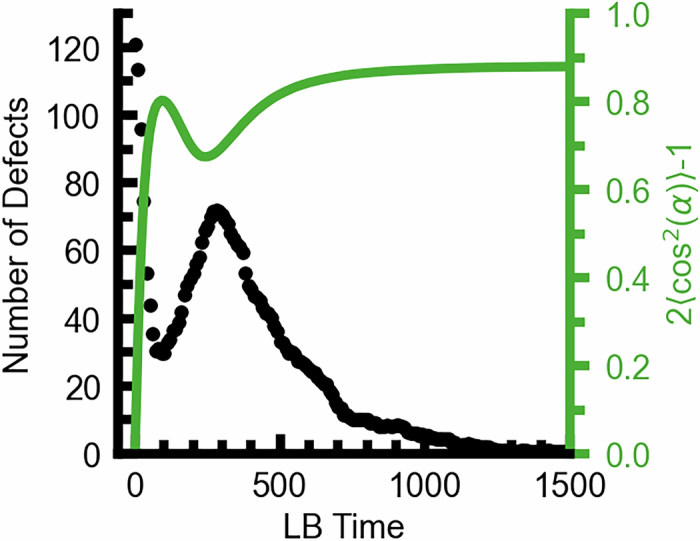
Number of defects with an AC field instead of constant external field. Non-monotonic coarsening kinetics is recovered when the external field is an AC field.

The model qualitatively reproduces the key experimental observations by relying on two central assumptions: (1) active stress drives the local nucleation of topological defects and (2) an external driving field induces global alignment. These mechanisms, implemented through the simplest symmetry-allowed forms of active stress and aligning fields, are sufficient to generate the non-monotonic coarsening dynamics observed in experiments. The resulting interplay between local activity and global ordering governs the spatiotemporal behaviour of endothelial cell layers and gives rise to a phase diagram with distinct tissue response regimes. This framework offers a mechanistic explanation rooted in competing timescales and coexisting symmetries. We note that when the external field is applied as an alternating current (AC) field in simulations, similar coarsening dynamics are observed (Extended Data Fig. 9) when the AC timescale is faster than the other relevant timescales. Although the model successfully captures the qualitative features of the experimental system, a quantitative comparison would require a more precise mapping between the model’s active stress and the stress generated by endothelial cell collectives across different densities, as well as the incorporation of mechanosensitive shear flow sensing.

Furthermore, this mechanistic explanation allows us to identify the interplay, which gives rise to defects that are not fully localized; rather, they are connected by string excitations in endothelial layers and are the biological analogues of the pair superfluid phase predicted for two-dimensional antiferromagnetic condensates under a magnetic field^56^. In this context, string excitations have been identified in classic XY models of magnetism, which possess both nematic and polar interactions^55,56,61^ and are characterized by a linear interaction potential, in addition to the normal logarithmic Coulomb potential between the defects giving rise to additional dynamics. These excitations have also been observed in the recently discovered ferroelectric nematic phase of matter^62–64^. The cell line studied in this work comes from the vascular system, where shear stress in a specific direction is paramount for healthy individuals as reversing shear stress has been associated with pathological conditions such as atherosclerosis^65^. However, the interplay between a preferred direction and active ordering could have widespread implications to many biological phenomena, for example, active ordering interacts with a preferred direction set by the axis of fore–aft symmetry during embryo development or during wound healing in which the geometry of the wound sets the preferred direction^66^. In addition, the intermediate stages are often essential in biological systems with a preferred direction, particularly in developmental processes in which they orchestrate cell fate decisions and tissue dynamics. For example, during embryogenesis, cells undergo transitions between mesenchymal and epithelial phenotypes, a key mechanism in cell lineage differentiation and organ development. Furthermore, transient morphogen gradients established during morphogenesis are critical in mediating tissue polarity and pattern formation.

The recent identification of a nematic phase during embryogenesis across multiple species, along with the observed presence of nematic defects during this process, highlights the potential importance of topological defects in development^67^. Our findings may set a benchmark for a wide range of biological phenomena in morphogenesis, tissue remodelling and disease progression, where spatiotemporally localized mechanical effects have consequences over long distances and timescales.

## Methods

### Primary HAOEC culture

Primary HAOECs (PeloBiotech, PB-CH-180-2011) were cultured in gelatine-coated T75 flasks (0.2% gelatine in phosphate-buffered saline incubated for 1 h at 37 °C) using the Endothelial Cell Growth Medium Kit Classic (PeloBiotech, PB-MH-100-2190) from passage 5 up to passage 9. For shear flow experiments, μ-slide I Luer ibiTreat (channel height, 0.2 mm) were incubated for 1 h with 40 μg ml^−1^ of collagen at 37 °C. After three washing steps with phosphate-buffered saline, 50 μl of HAOECs at a concentration of 3 × 10^6^ cells ml^−1^ up to 6 × 10^6 ^cells ml^−1^ were seeded into the flow channel and left at 37 °C for 1 h to allow them to form first adhesions. Then, the cell culture medium was added to the reservoirs, and the HAOECs were left overnight to fully adhere to the substrate. The confluent cell layer resulted in 12,000 up to 40,000 cells per a field of view of 40 mm^2^, with negligible cell proliferation during experiments (Extended Data Fig. 1d). The cell culture medium, phosphate-buffered saline and the flow channel slides were kept in the incubator 1 day before cell seeding to prevent bubble formation in the flow channels. Cell–cell contact between seeded cell colonies and cell layers formed through proliferation appeared comparable with no noticeable distinctions.

### Perfusion cell culture

To avoid bubble formation, the cell culture medium and perfusion set were kept in the incubator 1 day before the flow experiment. The ibidi pump system and perfusion set (ORANGE) were prepared according to the manufacturer’s instructions. After the pump system was calibrated, the flow channel was connected. To allow the cells to adapt to the shear stress, a flow program slowly increased the flow velocity until reaching the desired shear stress of 15 dyn cm^−2^ after 1 h. Throughout the experiments, cells were observed to migrate with and against the shear flow (Supplementary Video 6).

### Live-cell imaging

Phase contrast imaging was performed using a Leica DMi8 Thunder Imager and a ×10 (numerical aperture, 0.32) air objective. To gather sufficient statistical data, we stitched high-resolution images over a scale of several millimetres for up to 64 h. The area of the resulting field of view was 40 mm^2^. The ibidi stage top incubation system was adapted for live-cell imaging with a custom-built lid to accommodate the tubing. The fluidic unit of the pump system was kept in a nearby incubator at 37 °C and 5% CO_2_. Through a small opening in the rear of the incubator, the flow channel was mounted on the microscope stage and then connected to the pump system. Images were acquired every 20 min over at least 2.5 days.

### Characterization of nematic director field

We extracted the nematic field from the phase contrast images using a custom Python3 script based on the method from ref. ^30^ and as previously described in ref. ^31^. We computed the local nematic director **n** = **t** ⊗ **t** using the tangent vector **t** = (*I*_*y*_, –*I*_*x*_) to the gradient of the intensity of the image ∇*I*(*x*, *y*) = (*I*_*x*_, *I*_*y*_) within a box of dimensions *L* × *L*. From the local nematic director, we extracted the eigenvalues *n*_1_ > *n*_2_ and the corresponding eigenvector **e**_1_ to the biggest eigenvalue, which provided the nematic director associated with the pixel in the centre of the box. Here we used a box of 130 μm × 130 μm to compute the nematic director for a pixel. The nematic director field was computed for each pixel, and the spacing between two pixels is ~5.2 μm. We found that the cellular alignment dynamics are sensitive to both size and location of the field of view. In particular, smaller fields of view (<500 μm × 500 μm) exhibit variability in the observed behaviour. However, for fields of view of approximately 500 μm × 500 μm or larger, all six analysed regions displayed consistent non-monotonic alignment dynamics.

### Detection of nematic defects

We obtained the *Q* tensor from the nematic director field. The defect cores were then identified as the local maxima of the charge density $q=\frac{1}{4\uppi }({\partial }_{x}{Q}_{xk}{\partial }_{y}{Q}_{yk}-{\partial }_{x}{Q}_{yk}{\partial }_{y}{Q}_{xk})$, where ∣*q*∣ > 0.1. Only every fifth pixel in the nematic director field was considered for defect detection. The proliferation of cells seemed to be uncorrelated to the defect position (Extended Data Fig. 6e).

### Statistical analysis

Cell alignment dynamics under shear stress ranging from 15 dyn cm^−2^ to 20 dyn cm^−2^ was assessed in nine independent experiments. Monotonous alignment dynamics were observed in three independent experiments. Non-monotonous alignment dynamics were observed in six independent experiments. The 95% confidence intervals were computed using 1,000 bootstrapping iterations.

### Extraction of nearest-neighbour distance

Defects were considered for the *k*th-nearest-neighbour analysis if the defects were present for at least 4 h. We used trackpy to obtain and filter the defect trajectories^53^. From the defect trajectories, we constructed the *k*th-nearest-neighbour distance matrix, which we used to extract the *k*th-nearest-neighbour distance for *k* = 0, 1, 2 and 3.

### String visualization in experiments

The nematic director **n** = [cos(*α*), sin(*α*)] was defined in both orientation regions 0 ≤ *α*_1_ ≤ π (first and second quadrants) and –π/2 ≤ *α*_2_ ≤ π/2 (first and fourth quadrants). The parallel and perpendicular strings were defined to go between two resolution points if the absolute angle difference ∣Δ*α*_1_∣ or ∣Δ*α*_2_∣ was larger than π/2, respectively.

### Length measurement for parallel and perpendicular strings

From the absolute angle difference obtained for string visualization, a binary image was created by setting the points with ∣Δ*α*_1_∣ or ∣Δ*α*_2_∣ larger than π/2 to one and any other point to zero. Eventually, the string length was measured by obtaining the skeletons from the segmented features.

### Correlation functions

Taking the projection of the orientation parallel to the preferred axis *δn* (Extended Data Fig. 3a) allowed us to identify how far away the director was from the preferred axis. Using this, we observed how regions of similar orientation coarsened by looking at the correlation functions *C*_*δn*⊥_(Δ*r*_∥_) (Extended Data Fig. 3b) and *C*_*δn*⊥_(Δ*r*_⊥_) (Extended Data Fig. 3c), where we split up the correlations along the perpendicular (Δ*r*_⊥_) and parallel (Δ*r*_∥_) direction to the preferred axis.

Comparing the correlation functions along the axes perpendicular (Δ*r*_⊥_) and parallel (Δ*r*_∥_) to the preferred orientation revealed that as the system evolved towards an aligned state, the discrepancy in the decay behaviour of the correlation functions became more pronounced (Extended Data Fig. 3d,e,g,h). This trend was observed in both experiments and simulations.

We extracted a length scale *ξ* from the correlation functions by determining the length when the correlation function reached the value 1/*ϵ*. In the experiments, the correlation along both axes became more persistent over time (Extended Data Fig. 3f). However, the perpendicular length *ξ*_⊥_ grew more monotonically, whereas the parallel length *ξ*_∥_ was strongly connected to the multiple phases. By contrast, in simulations, only the parallel axis showed a stronger correlation in the parallel direction, whereas the perpendicular axis remained unaffected (Extended Data Fig. 3i). This phenomenon arises as a secondary effect of epithelial cells breaking mirror symmetry (Extended Data Fig. 4). Previous studies have established that these endothelial cells can spontaneously rotate in either direction due to self-generated torques: *f*_*q*_ = 2*ωs*[−*Q*_*xy*_, *Q*_*xx*_; *Q*_*xx*_, *Q*_*xy*_] (ref. ^49^). Here *ω* is the magnitude of the spin and *s* sets the direction. This variation breaks the mirror symmetry, resulting in spreading along the *y* direction (Extended Data Fig. 5a). However, we emphasize that these rotational effects are secondary to the primary influence of the interplay between activity and the aligning field. This interplay governs the coarsening dynamics and the emergence of string-like structures, which are the main focus of this study. In the absence of activity, but with this torque, coarsening still proceeds monotonically.

### String visualization in simulations

The nematic director **n** = [cos(*α*), sin(*α*)] was defined in both orientation regions 0 ≤ *α*_1_ ≤ π (first and second quadrants) and –π/2 ≤ *α*_2_ ≤ π/2 (first and fourth quadrants). The parallel and perpendicular strings were defined to go between two resolution points if the absolute angle difference ∣Δ*α*_1_∣ or ∣Δ*α*_2_∣ was larger than π/2, respectively.

### Removal of shear flow

We conducted two additional experiments in which the flow was stopped at different points during the formation of global cell alignment (Extended Data Fig. 1e,f). If the shear flow stopped after near-perfect alignment (Extended Data Fig. 1e), we find no immediate disordering. However, the number of nematic defects slowly increased over time, suggesting that the system relaxes very slowly back to a disordered state in the absence of flow. Two independent experiments were carried out with the same experimental observations.

If the shear flow is removed at the start of the final steady-state ordering (Extended Data Fig. 1f), we find that the system quickly relaxed back to a disordered state, as the alignment was not yet well established. Two independent experiments were performed that yielded similar experimental observations.

These observations indicate that the relaxation dynamics are influenced by the degree of alignment at the moment the flow is stopped. A well-aligned system undergoes slow relaxation, whereas a partially aligned system relaxes more rapidly. This aligns with model predictions, as defect-nucleating instability develops more quickly when the system already exhibits substantial deformation.

### Effective Zeeman splitting of strings

In a typical nematic system, whether active or passive, all nematic orientations *α*, or isophase lines, are equivalent. However, the strings we highlight correspond to isophase lines that experience maximum and minimum energy costs due to the external field or shear flow.

This behaviour is analogous to Zeeman splitting and informs our comparison to superfluid pair behaviour, where the nematic symmetry of boson pairing interacts with the preferred direction imposed by a Zeeman field^56^. The two domain types we identify, which are misaligned relative to these isophase lines, experience the same energy cost (Extended Data Fig. 7c).

From this, we define a ground state without defects (Extended Data Fig. 7d) and a topologically excited defect-laden state (Extended Data Fig. 7e), where activity and energy either reinforce or counteract each other. When the velocity of a +1/2 defect is oriented such that its motion eliminates the perpendicular alignment of the director, we find that the annihilation of the defect pair would restore the defect-free orientation (Extended Data Fig. 7e; defect pair along the *x* axis). Consequently, these defect pairs annihilate rapidly.

Conversely, when the self-propelled velocity of a +1/2 defect is oriented in a manner such that +1/2 motion propagates along the disfavoured perpendicular alignment (Extended Data Fig. 7e; defect pair along the *y* axis), this configuration would hinder annihilation, thereby inducing frustration within the system. In this scenario, competition emerges between the active self-propulsion and the misalignment relative to the flow-aligning field, which serves to stabilize the defect pair. This stabilization results in the defect pairs becoming long-lived.

### Simulations

To complement the experiments, we simulated the cell layers as an active nematic film, where the third-dimensional shear flow was incorporated as an aligning field. We solved the continuum equations using a hybrid lattice Boltzmann approach^54^. We assumed that the active nematic film flows with collective velocity $\bf{u}$ and has long-range orientational order described by the tensor order parameter *Q*, which mimics the cell alignment.

The dynamics of the orientational order parameter *Q* at each position $\bf{r}$ and time *t* is described by the Beris–Edwards transport equation:

1 $$\left({\partial }_{t}+\bf{u}\cdot {\nabla}\right){{Q}}-{{S}}-{{{f}}}_{{{Q}}}={\Gamma }_{Q}{{H}}.$$

The co-rotation term $${{S}}=(\xi {\;{D}}+{{\Omega }})({{Q}}+\frac{1}{3}{{I}})+({{Q}}+\frac{1}{3}{{I}})(\xi {\;{D}}-{{\Omega }})$$$$-2\xi ({{Q}}+\frac{1}{3}{{I}})$$$${\rm{tr}}({{Q}}{{W}})$$ determines the alignment of the cells in response to gradients in the velocity field, where *Ω* is the rotational part and *D* is the extensional part of the velocity gradient tensor $${{W}}={\nabla}{\bf{u}}={{\Omega}}+{{D}}$$. The alignment parameter *ξ* is considered deep in the flow-aligning regime and set to *ξ* = 0.9 as cells align strongly with themselves. *f*_*Q*_ is a possible active spin or active torque, as discussed in the next section^49^.

The molecular field ${{H}}=-(\frac{\delta {\mathcal{F}}}{\delta {{Q}}}-\frac{1}{3}{{I}}\,{\rm{tr}}\frac{\delta {\mathcal{F}}}{\delta {{Q}}})$ is a functional derivative of free energy density ${\mathcal{F}}$, describing relaxation towards equilibrium at rate *Γ*_*Q*_. The free energy consists of a Landau–de Gennes contribution, ${f}_{{\rm{LdG}}}={A}_{0}\{\frac{1}{2}(1-\frac{\nu }{3}){\rm{tr}}[{{{Q}}}^{2}]-\frac{\nu }{3}{\rm{tr}}[{{{Q}}}^{3}]+\frac{\nu }{4}{\rm{tr}}{[{{{Q}}}^{2}]}^{2}\}$ with *A*_0_ = 0.05, and a Frank–Oseen deformation ${f}_{{\rm{FO}}}=\frac{K}{2}{({\nabla}{{Q}})}^{2}$ with *K* = 0.02. We used *ν* = 2.55, which favours the isotropic state in the absence of activity or any aligning field. Last, it contains a field strength *f*_Field_ = –*ϵ*_0_*E* × *Q* × *E*, where *E* is a matrix setting the direction of the flow and *ϵ*_0_ is the strength of the field that induces nematic ordering along the flow axis. We note that when the field is applied as an a.c. field, similar coarsening dynamics are observed (Extended Data Fig. 9).

The system also obeys the Navier–Stokes equations for the velocity field within the active film. Assuming constant fluid mass density *ρ* (not active material concentration *ϕ*) leads to the incompressibility condition

2 $${\nabla}\cdot {\bf{u}}=0$$

and

3 $${\rho} \left({\partial}_{t}+{\bf{u}}\cdot {\nabla}\right){\bf{u}}=-{\nabla} {{p}}+{\nabla}\cdot {{\pi}}-\gamma {\bf{u}},$$

where *p* is the pressure and *Π* is the stress tensor that includes the standard viscous stress *Π*^visc^ = 2*ηE* for film viscosity *η* = 2/3. Furthermore, it contains the elastic stress due to the nematic nature of the order parameter

4 $$\begin{array}{rcl}{\pi}^{\rm{elastic}}&=&2\xi {{{\mathcal{Q}}}}({{Q}}:{{H}})-\xi\;{{H}}\cdot {{{\mathcal{Q}}}}-\xi {{{\mathcal{Q}}}}\cdot {{H}}\\ &&-{\nabla}{{Q}}:\frac{{\delta} {\mathcal{F}}}{{\delta} {\nabla}{{Q}}}+{{Q}}\cdot {{H}}-{{H}}\cdot {{Q}},\end{array}$$

where ${{{\mathcal{Q}}}}={{Q}}+{{I}}/3$. Last, the stress contains an active component

5 $${{{\pi }}}^{{\rm{act}}}=-\zeta {{Q}}.$$

We used *ζ* = 0.03 to induce weak nematic ordering in the absence of an aligning field. The activity is assumed to be extensile, as the coarse-grained activity of the entire cell monolayer, derived from the motion direction of defects in the absence of flow (Extended Data Fig. 6a–d), exhibits a nematic character, in contrast to the contractile nature of individual cells^68,69^.

### Active torque

We noted that here the coarsening dynamics along the perpendicular direction are less pronounced compared with the experimental measurements. The reason for this discrepancy is that in the experiments, one of the two domains tends to dominate over time (Extended Data Fig. 4). The domain that dominates appears to differ between different realizations of the same experiment. This variation breaks the mirror symmetry, resulting in spreading along the *y* direction.

In agreement with this observation, previous work has well established that endothelial cells can spontaneously rotate (which can occur in either direction) through self-generated torques^49^. To capture this, we introduced an additional active torque *f*_*q*_ = 2*ωs*[−*Q*_*xy*_, *Q*_*xx*_; *Q*_*xx*_, *Q*_*xy*_] into the evolution equation for the nematic tensor. With this additional term, we capture the growth of domains along the perpendicular *y* direction (Extended Data Fig. 5), as well as the stronger alignment along the *x* axis (Extended Data Fig. 5b–f), improving the match between experiments and simulations. In particular, we observe that in both experiments and simulations, for the perpendicular spatial component of the correlation function (*C*_*δn*⊥_(Δ*r*_⊥_)), the value of the long-range correlation increases over time, whereas the decay length remains the same. By contrast, for the parallel spatial component *C*_*δn*⊥_(Δ*r*_∥_), now both long-range correlation value and correlation length increase substantially with time.

Such rotation effects are secondary to the impact from the interplay between activity and aligning field: the interplay sets the coarsening dynamics and defect structure. Without activity, coarsening occurs monotonically. The rotation acts as a secondary effect through biasing the long-time orientation field towards one domain by breaking the mirror symmetry. Indeed, numerical simulations confirm that this torque alone cannot produce non-monotonic coarsening, and when the normal activity is zero, we observe monotonic kinetics (Extended Data Fig. 5g). For the rest of the manuscript, we ignore this secondary affect.

### Timescales

The active nematic with an external field exhibits three relevant timescales. The first is the active nematic timescale, given by the ratio of viscosity to activity, ${\tau }_{\zeta }=\frac{\eta }{\zeta }$ (ref. ^37^). The second is a nematic coarsening timescale, determined by the intrinsic nematic ordering, which scales with rotational viscosity $\frac{1}{\varGamma }$ and the interaction strength of defects, *A*_0_, leading to ${\tau }_{n}=\frac{1}{\varGamma {A}_{0}}$ (ref. ^37^). The third is set by the external field strength *ϵ*_0_, yielding ${\tau }_{e}=\frac{1}{\varGamma {\epsilon }_{0}}$.

Using the standard values provided in the ‘Simulations’ section, one finds *τ*_e_ ≈ 20, *τ*_*ζ*_ ≈ 33 and *τ*_n_ ≈ 200, all in simulation lattice Boltzmann time units. Therefore, simple nematic coarsening occurs when the external timescale *τ*_e_ is the shortest. Subsequently, the active nematic timescale sets in, leading to the nucleation of defects. On longer timescales, the orientation field dominates.

In the phase diagram shown in Fig. 2, increasing the activity corresponds to a reduction in the active timescale, *τ*_*ζ*_. Similarly, increasing the external field’s strength corresponds to a reduction in the external timescale, *τ*_e_. Hence, monotonic ordering is observed when *τ*_e_ < *τ*_*ζ*_ and *τ*_n_ < *τ*_*ζ*_, whereas active turbulence arises when *τ*_e_ > *τ*_*ζ*_ and *τ*_n_ > *τ*_*ζ*_.

Here we linked the timescale of the intermediate disordering phase to the active nematic timescale. This can be considered the timescale for defects to appear and annihilate in cell monolayers. In experimental systems, this timescale is dependent and can be varied through a variety of factors, including substrate stiffness and cell–cell interactions as well as individual cell contractility^36,38^.

## Online content

Any methods, additional references, Nature Portfolio reporting summaries, source data, extended data, supplementary information, acknowledgements, peer review information; details of author contributions and competing interests; and statements of data and code availability are available at 10.1038/s41567-025-03014-4.

## Supplementary information

Supplementary Video 1 Phase contrast images of HAOECs exposed to 20 dyn cm^−2^ shear stress flowing from left to right. The HAOECs align in the flow direction.

Supplementary Video 2 Phase contrast images of HAOECs from Supplementary Video 1, with an overlay of the nematic director field and the topological defects as the cells reorient in the flow direction.

Supplementary Video 3 Simulations of the nematic director field, coloured by domain, were conducted with an anisotropic field strength of *ν* = 0.25 along the horizontal axis and an activity set to *ζ* = 0.03.

Supplementary Video 4 Phase contrast images of HAOECs with an overlay of the domains. The defects are connected by red and green strings, indicating the energy-intensive transition and the energy-efficient transition between the domains.

Supplementary Video 5 Simulations of the nematic director, coloured by domain, from Supplementary Video 3, with an overlay of defects that are connected by red and green strings indicating the energy-intensive transition and energy-efficient transition between the domains, respectively.

Supplementary Video 6 Tracked cell velocity along the sheared axis.

Supplementary Video 7 Tracked cell velocity (arrows) with the local cell orientation (lines) overlaid on top of the tracked cell velocity along the sheared axis.

## Data Availability

Modelling source and all experimental data that support the plots are provided with this Article and also available via Zenodo at 10.5281/zenodo.15322247 (ref. ^70^). The raw data obtained for this study are available from the corresponding authors upon reasonable request

## Code availability

The main program codes can be downloaded from the data repository at 10.5281/zenodo.15322247 (ref. ^70^). Other programs are available from the corresponding authors upon request.

## References

[1] Resnick, N. et al. Fluid shear stress and the vascular endothelium: for better and for worse. *Prog. Biophys. Mol. Biol.* **81**, 177–199 (2003). 12732261 10.1016/s0079-6107(02)00052-4

[2] Virchow, R. *Die Cellularpathologie in ihrer Begründung auf physiologische und pathologische Gewebelehre* 3rd edn (Verlag von August Hirschwald, 1859).

[3] Eskin, S. G., Ives, C. L., McIntire, L. V. & Navarro, L. T. Response of cultured endothelial cells to steady flow. *Microvasc. Res.* **28**, 87–94 (1984). 6748961 10.1016/0026-2862(84)90031-1

[4] Galbraith, C., Skalak, R. & Chien, S. Shear stress induces spatial reorganization of the endothelial cell cytoskeleton. *Cell Motil.* **40**, 317–330 (1998). 10.1002/(SICI)1097-0169(1998)40:4<317::AID-CM1>3.0.CO;2-8 9712262

[5] Barakat, A. I. & Lieu, D. K. Differential responsiveness of vascular endothelial cells to different types of fluid mechanical shear stress. *Cell Biochem. Biophys.* **38**, 323–343 (2003). 12794271 10.1385/cbb:38:3:323

[6] Jafari, A., Behjat, E., Malektaj, H. & Mobini, F. Alignment behavior of nerve, vascular, muscle, and intestine cells in two-and three-dimensional strategies. *WIREs Mech. Dis.* **15**, e1620 (2023). 37392045 10.1002/wsbm.1620

[7] Anon, E. et al. Cell crawling mediates collective cell migration to close undamaged epithelial gaps. *Proc. Natl Acad. Sci. USA* **109**, 10891–10896 (2012). 22711834 10.1073/pnas.1117814109 PMC3390890

[8] Aigouy, B. et al. Cell flow reorients the axis of planar polarity in the wing epithelium of drosophila. *Cell* **142**, 773–786 (2010). 20813263 10.1016/j.cell.2010.07.042

[9] Maroudas-Sacks, Y. et al. Topological defects in the nematic order of actin fibres as organization centres of hydra morphogenesis. *Nat. Phys.* **17**, 251–259 (2021).

[10] Duclos, G., Garcia, S., Yevick, H. G. & Silberzan, P. Perfect nematic order in confined monolayers of spindle-shaped cells. *Soft Matter* **10**, 2346–2353 (2014). 24623001 10.1039/c3sm52323c

[11] Comelles, J. et al. Epithelial colonies in vitro elongate through collective effects. *eLife* **10**, e57730 (2021). 33393459 10.7554/eLife.57730 PMC7850623

[12] Saw, T. B. et al. Topological defects in epithelia govern cell death and extrusion. *Nature* **544**, 212–216 (2017). 28406198 10.1038/nature21718 PMC5439518

[13] Kawaguchi, K., Kageyama, R. & Sano, M. Topological defects control collective dynamics in neural progenitor cell cultures. *Nature* **545**, 327–331 (2017). 28403137 10.1038/nature22321

[14] Guillamat, P., Blanch-Mercader, C., Pernollet, G., Kruse, K. & Roux, A. Integer topological defects organize stresses driving tissue morphogenesis. *Nat. Mater.* **21**, 588–597 (2022). 35145258 10.1038/s41563-022-01194-5 PMC7612693

[15] Tang, Y., Chen, S., Bowick, M. J. & Bi, D. Cell division and motility enable hexatic order in biological tissues. *Phys. Rev. Lett.* **132**, 218402 (2024). 38856284 10.1103/PhysRevLett.132.218402 PMC11267118

[16] Kosterlitz, J. M. Nobel lecture: Topological defects and phase transitions. *Rev. Mod. Phys.* **89**, 040501 (2017).

[17] Kosterlitz, J. M. & Thouless, D. J. Ordering, metastability and phase transitions in two-dimensional systems. *J. Phys. C Solid State Phys.* **6**, 1181–1203 (1973). 10.1088/0953-8984/28/48/481001 27665689

[18] Kosterlitz, J. M. The critical properties of the two-dimensional *XY* model. *J. Phys. C Solid State Phys.* **7**, 1046–1060 (1974).

[19] Sethna, J. P. Order parameters, broken symmetry, and topology. Preprint at https://arxiv.org/abs/cond-mat/9204009 (1992).

[20] Liu, M., Nam, H., Kim, J., Fiete, G. A. & Shih, C.-K. Influence of nanosize hole defects and their geometric arrangements on the superfluid density in atomically thin single crystals of indium superconductor. *Phys. Rev. Lett.* **127**, 127003 (2021). 34597098 10.1103/PhysRevLett.127.127003

[21] Nowak, W. et al. Effect of the presence of structural defects on the superconducting properties of (NbTa)_0.67_(MoHfW)_0.33_ and Nb-47wt%Ti. *Metals* **13**, 1779 (2023).

[22] Akhtar, R., Sherratt, M. J., Cruickshank, J. K. & Derby, B. Characterizing the elastic properties of tissues. *Mater. Today* **14**, 96–105 (2011). 10.1016/S1369-7021(11)70059-1 PMC3378034 22723736

[23] Dessalles, C. A., Leclech, C., Castagnino, A. & Barakat, A. I. Integration of substrate- and flow-derived stresses in endothelial cell mechanobiology. *Commun. Biol.* **4**, 764 (2021). 34155305 10.1038/s42003-021-02285-w PMC8217569

[24] Dolan, J. M., Meng, H., Singh, S., Paluch, R. & Kolega, J. High fluid shear stress and spatial shear stress gradients affect endothelial proliferation, survival, and alignment. *Ann. Biomed. Eng.* **39**, 1620–1631 (2011). 21312062 10.1007/s10439-011-0267-8 PMC4809045

[25] Viggers, R. F., Wechezak, A. R. & Sauvage, L. R. An apparatus to study the response of cultured endothelium to shear stress. *J. Biomech. Eng.* **108**, 332–337 (1986). 3795878 10.1115/1.3138624

[26] Baeyens, N. et al. Vascular remodeling is governed by a VEGFR3-dependent fluid shear stress set point. *eLife* **2015**, e04645 (2015). 10.7554/eLife.04645 PMC4337723 25643397

[27] Ostrowski, M. A. et al. Microvascular endothelial cells migrate upstream and align against the shear stress field created by impinging flow. *Biophys. J.* **106**, 366–374 (2014). 24461011 10.1016/j.bpj.2013.11.4502 PMC3907231

[28] Krüger-Genge, A., Blocki, A., Franke, R. P. & Jung, F. Vascular endothelial cell biology: an update. *Int. J. Mol. Sci.* **20**, 4411 (2019). 31500313 10.3390/ijms20184411 PMC6769656

[29] Makwana, O. et al. Human aortic endothelial cells respond to shear flow in well-plate microfluidic devices. *Altern. Lab. Anim.* **45**, 177–190 (2017). 28994298 10.1177/026119291704500407

[30] Boudaoud, A. et al. Fibriltool, an imagej plug-in to quantify fibrillar structures in raw microscopy images. *Nat. Protoc.* **9**, 457–463 (2014). 24481272 10.1038/nprot.2014.024

[31] Sciortino, A. et al. Polarity and chirality control of an active fluid by passive nematic defects. *Nat. Mater.* **22**, 260–268 (2023). 36585435 10.1038/s41563-022-01432-w PMC9894751

[32] Yurke, B., Pargellis, A., Kovacs, T. & Huse, D. Coarsening dynamics of the *XY* model. *Phys. Rev. E* **47**, 1525 (1993). 10.1103/physreve.47.1525 9960172

[33] Kinoshita, Y. & Uchida, N. Flow patterns and defect dynamics of active nematic liquid crystals under an electric field. *Phys. Rev. E* **108**, 014605 (2023). 37583184 10.1103/PhysRevE.108.014605

[34] Mueller, R., Yeomans, J. M. & Doostmohammadi, A. Emergence of active nematic behavior in monolayers of isotropic cells. *Phys. Rev. Lett.* **122**, 048004 (2019). 30768306 10.1103/PhysRevLett.122.048004

[35] Lin, S.-Z., Merkel, M. & Rupprecht, J.-F. Structure and rheology in vertex models under cell-shape-dependent active stresses. *Phys. Rev. Lett.* **130**, 058202 (2023). 36800465 10.1103/PhysRevLett.130.058202

[36] Chiang, M., Hopkins, A., Loewe, B., Marchetti, M. C. & Marenduzzo, D. Intercellular friction and motility drive orientational order in cell monolayers. *Proc. Natl Acad. Sci. USA* **121**, e2319310121 (2024). 39302997 10.1073/pnas.2319310121 PMC11459176

[37] Hemingway, E. J., Mishra, P., Marchetti, M. C. & Fielding, S. M. Correlation lengths in hydrodynamic models of active nematics. *Soft Matter* **12**, 7943–7952 (2016). 27722646 10.1039/c6sm00812g

[38] Bera, P. K., McCord, M., Zhang, J. & Notbohm, J. Energy dynamics powered by traction and stress control formation and motion of +1/2 topological defects in epithelial cell monolayers. Preprint at https://arxiv.org/abs/2501.04827v1 (2025). 10.1016/j.newton.2025.100231 PMC12416539 40926784

[39] Trepat, X. et al. Physical forces during collective cell migration. *Nat. Phys.* **5**, 426–430 (2009).

[40] Kadohama, T. et al. p38 mitogen-activated protein kinase activation in endothelial cell is implicated in cell alignment and elongation induced by fluid shear stress. *Endothelium* **13**, 43–50 (2006). 16885066 10.1080/10623320600660219

[41] Leclech, C. et al. Topography-induced large-scale antiparallel collective migration in vascular endothelium. *Nat. Commun.* **13**, 2797 (2022). 35589751 10.1038/s41467-022-30488-0 PMC9120158

[42] Lacroix, M. et al. Emergence of bidirectional cell laning from collective contact guidance. *Nat. Phys.* **20**, 1324–1331 (2024).

[43] Thijssen, K. et al. Submersed micropatterned structures control active nematic flow, topology, and concentration. *Proc. Natl Acad. Sci. USA* **118**, e2106038118 (2021). 34535551 10.1073/pnas.2106038118 PMC8463884

[44] Lawson-Keister, E. & Manning, M. L. Jamming and arrest of cell motion in biological tissues. *Curr. Opin. Cell Biol.* **72**, 146–155 (2021). 34461581 10.1016/j.ceb.2021.07.011

[45] Giomi, L. & Marchetti, M. C. Polar patterns in active fluids. *Soft Matter* **8**, 129–139 (2012).

[46] Blanch-Mercader, C., Guillamat, P., Roux, A. & Kruse, K. Quantifying material properties of cell monolayers by analyzing integer topological defects. *Phys. Rev. Lett.* **126**, 028101 (2021). 33512187 10.1103/PhysRevLett.126.028101

[47] Adar, R. M. & Joanny, J.-F. Active-gel theory for multicellular migration of polar cells in the extra-cellular matrix. *New J. Phys.* **24**, 073001 (2022).

[48] Dedenon, M. et al. Density-polarity coupling in confined active polar films: asters, spirals, and biphasic orientational phases. *Phys. Rev. Lett.* **131**, 268301 (2023). 38215373 10.1103/PhysRevLett.131.268301

[49] Li, Z.-Y., Zhang, D.-Q., Lin, S.-Z. & Li, B. Pattern formation and defect ordering in active chiral nematics. *Phys. Rev. Lett.* **125**, 098002 (2020). 32915620 10.1103/PhysRevLett.125.098002

[50] Pargellis, A. N., Green, S. & Yurke, B. Planar *XY*-model dynamics in a nematic liquid crystal system. *Phys. Rev. E* **49**, 4250 (1994). 10.1103/physreve.49.4250 9961717

[51] Dutta, S. & Roy, S. K. Persistence exponents and scaling in two-dimensional *XY* model and a nematic model. *J. Phys. A Math. Gen.* **38**, 5859 (2005).

[52] Shankar, S., Ramaswamy, S., Marchetti, M. C. & Bowick, M. J. Defect unbinding in active nematics. *Phys. Rev. Lett.* **121**, 108002 (2018). 30240234 10.1103/PhysRevLett.121.108002

[53] Allan, D. B., Caswell, T., Keim, N. C., van der Wel, C. M. & Verweij, R. W. soft-matter/trackpy: v0.6.1. *Zenodo*10.5281/zenodo.7670439 (2023).

[54] Thampi, S. & Yeomans, J. Active turbulence in active nematics. *Eur. Phys. J. Spec. Top.* **225**, 651–662 (2016).

[55] Lee, D. & Grinstein, G. Strings in two-dimensional classical *XY* models. *Phys. Rev. Lett.* **55**, 541 (1985). 10032380 10.1103/PhysRevLett.55.541

[56] James, A. & Lamacraft, A. Phase diagram of two-dimensional polar condensates in a magnetic field. *Phys. Rev. Lett.* **106**, 140402 (2011). 21561170 10.1103/PhysRevLett.106.140402

[57] Turiv, T. et al. Polar jets of swimming bacteria condensed by a patterned liquid crystal. *Nat. Phys.* **16**, 481–487 (2020).

[58] Thijssen, K., Metselaar, L., Yeomans, J. M. & Doostmohammadi, A. Active nematics with anisotropic friction: the decisive role of the flow aligning parameter. *Soft Matter* **16**, 2065–2074 (2020). 32003382 10.1039/c9sm01963d

[59] Metaxa, E. et al. Nitric oxide-dependent stimulation of endothelial cell proliferation by sustained high flow. *Am. J. Physiol. Heart Circ. Physiol.* **295**, 736–742 (2008). 10.1152/ajpheart.01156.2007 PMC2519227 18552158

[60] Hahn, C., Wang, C., Orr, A. W., Coon, B. G. & Schwartz, M. A. Jnk2 promotes endothelial cell alignment under flow. *PLoS ONE* **6**, e24338 (2011). 21909388 10.1371/journal.pone.0024338 PMC3164210

[61] Shi, Y., Lamacraft, A. & Fendley, P. Boson pairing and unusual criticality in a generalized *XY* model. *Phys. Rev. Lett.* **107**, 240601 (2011). 22242982 10.1103/PhysRevLett.107.240601

[62] Lavrentovich, O. D. Ferroelectric nematic liquid crystal, a century in waiting. *Proc. Natl Acad. Sci. USA* **117**, 14629–14631 (2020). 32541021 10.1073/pnas.2008947117 PMC7334533

[63] Chen, X. et al. First-principles experimental demonstration of ferroelectricity in a thermotropic nematic liquid crystal: polar domains and striking electro-optics. *Proc. Natl Acad. Sci. USA* **117**, 14021–14031 (2020). 32522878 10.1073/pnas.2002290117 PMC7322023

[64] Ma, Z. et al. Half-integer topological defects paired via string micelles in polar liquids. *PNAS Nexus* **3**, pgae552 (2024). 39703229 10.1093/pnasnexus/pgae552 PMC11658416

[65] Conway, D. E., Eskin, S. G. & McIntire, L. V. in *Biomaterials Science: An Introduction to Materials (Third Edition)* 474–482 (Elsevier, 2013).

[66] Cohen, D. J., Gloerich, M. & Nelson, W. J. Epithelial self-healing is recapitulated by a 3D biomimetic e-cadherin junction. *Proc. Natl Acad. Sci. USA* **113**, 14698–14703 (2016). 27930308 10.1073/pnas.1612208113 PMC5187740

[67] Li, X. et al. Emergence of cellular nematic order is a conserved feature of gastrulation in animal embryos. *Nat. Commun.* **16**, 5946 (2025). 40595575 10.1038/s41467-025-61045-0 PMC12217158

[68] Killeen, A., Bertrand, T. & Lee, C. F. Polar fluctuations lead to extensile nematic behavior in confluent tissues. *Phys. Rev. Lett.* **128**, 078001 (2022). 35244433 10.1103/PhysRevLett.128.078001

[69] Nejad, M. R. et al. Stress-shape misalignment in connt cell layers. *Nat. Commun.* **15**, 3628 (2024). 38684651 10.1038/s41467-024-47702-w PMC11059169

[70] Ruider, I. et al. String and topological defects govern ordering kinetics in endothelial cell layers. Zenodo 10.5281/zenodo.15322247 (2025). 10.1038/s41567-025-03014-4 PMC12518141 41098542

